# Failure to Thrive in Infants with Prader-Willi Syndrome and Long-Term Effects of GH Treatment on FTT

**DOI:** 10.1159/000553147

**Published:** 2026-08-24

**Authors:** Demi J. Trueba-Timmermans, Gerthe F. Kerkhof, Lionne N. Grootjen, Edmond H.H.M. Rings, Anita C.S. Hokken-Koelega

**Affiliations:** a Dutch Reference Centre for Prader-Willi Syndrome, Rotterdam, The Netherlands; b Dutch Growth Research Foundation, Rotterdam, The Netherlands; c Department of Pediatrics, Subdivision of Endocrinology, Erasmus University Medical Centre-Sophia Children’s Hospital, Rotterdam, The Netherlands; d Department of Pediatrics, Erasmus University Medical Centre-Sophia Children’s Hospital, Rotterdam, The Netherlands

**Keywords:** Prader-Willi syndrome, Failure to thrive, Infants, Growth hormone

## Abstract

**Introduction:**

Failure to thrive (FTT) with feeding difficulties and hypotonia are well-known features in infants with Prader-Willi syndrome (PWS). Limited studies reported the percentage of infants with FTT and it is unknown whether growth hormone (GH) treatment affects body composition of infants with or without FTT differently. Aims of the study were to evaluate the percentage of infants with PWS with FTT from birth to age 6 months and to investigate the 5-year GH effects on body composition in infants with or without FTT at GH start.

**Methods:**

We defined FTT as weight-for-length <−2 SDS and evaluated the percentage of infants with FTT in a group of 54 infants from birth to age 6 months. In addition, we investigated in 166 children the 5-year GH effects on body composition, comparing children with and without FTT at GH start. All infants were treated with 1.0 mg GH/m2/day (∼0.035 mg/kg/day). Body composition was determined by dual-energy X-ray absorptiometry.

**Results:**

From birth to age 6 months, 29% of infants had FTT at 1 month, 20% at 3 months, and 16% at 6 months. At GH start, at a median age of 1.03 (IQR 0.45, 1.89) years, 12% (20/166) had FTT (FTT_GH start_). Infants with FTT_GH start_ had a lower lean body mass index (LBMI) compared to non-FTT_GH start_, but a greater catch-up in LBMI in the first year of GH, resulting in a similar LBMI as the non-FTT_GH start_ after 5 years of GH treatment. Children with FTT_GH start_ had lower FM% after 5 years of GH treatment, but this difference disappeared after including covariates.

**Conclusions:**

Twenty-nine percent of infants had FTT at 1 month, 20% at 3 months, and 16% at 6 months. Twelve percent had FTT at the start of GH treatment. Children with FTT at GH start showed greatest LBM improvement during GH treatment, resulting in a similar LBM after 5 years of GH treatment as those without FTT at GH start.

## Introduction

Prader-Willi syndrome (PWS) is a rare syndrome caused by the lack of expression of the paternally derived chromosome 15, due to a paternal deletion or maternal uniparental disomy, and in rare cases by an imprinting centre defect or paternal chromosomal translocation [1, 2]. The syndrome is characterized by muscular hypotonia, short stature, developmental delay, hyperphagia, and an abnormal body composition with a high body fat percentage and low lean body mass (LBM) [2].

Full-term birthweight, length, and BMI are about 15–20% lower in neonates with PWS than in their siblings [3]. In addition, neonates with PWS have a lower birthweight compared to healthy peers [4]. Subsequently, failure to thrive (FTT) with feeding difficulties and severe hypotonia are prominent [3]. The feeding problems in early life are partly caused by the hypotonia, but the additional lack of appetite also plays a role [3, 5]. Most neonates with PWS need some type of assisted feeding, such as nasogastric tube feeding, until the age of 3–9 months to prevent FTT. Miller et al. [3] identified different nutritional phases of PWS during childhood, and during the second nutritional phase, the feeding difficulties gradually improve and most infants with PWS grow steadily along a growth percentile with weight increasing at a normal rate [1]. In most children, body weight starts to increase between 18 and 36 months of age, without an increase in calorie intake or interest in food. Thereafter, during early childhood, the appetite and interest in food start to increase abnormally [3]. This stage often evolves into a state of hyperphagia, with food-seeking behaviour and the onset of obesity when children are left unattended.

There is no consensus which specific anthropometric criterion should be used to define FTT. The WHO guideline defines FTT in infants and children under 5 years of age as having a weight-for-height or weight-for-length z score below 2 standard deviations of the median [6]. Supporting definitions include weight for age, body mass index for age, and height for age below −2 standard deviations [7, 8].

Few studies investigated the prevalence of FTT in infants with PWS. Miller et al. [3] described that 9 of 58 (16%) infants had severe, prolonged FTT despite receiving adequate calories during the first nutritional phase (age 0–9 months). In another study, 6 of the 9 (67%) infants, aged 10 days to 11 months, were underweight [9].

Abnormal body composition is already present at birth in children with PWS with or without FTT [10]. Studies have shown that GH treatment in children with PWS improves body composition, leading to less fat mass and more LBM, linear growth, and physical strength [11–16]. However, the effect of 5 years of GH treatment on body composition in children with PWS and FTT at start of GH treatment compared to those without FTT has not yet been described.

The primary aim of our study was to investigate the percentage of infants with FTT in infants with PWS before the start of GH treatment. We hypothesized that the percentage of infants with FTT would be 15–20% in young infants with PWS and that this percentage would decline with age. The other primary aim was to investigate the effect of GH treatment on body composition in infants with FTT at GH start (FTT_GH start_) compared to infants without FTT at GH start (non-FTT_GH start_). We previously showed that infants with low motor development had a greater improvement in motor development during GH treatment [12]. We, therefore, hypothesized that infants with FTT at start of GH treatment would have a lower lean body mass index (LBMI) but show greater catch-up in LBMI compared to those without FTT. Our secondary aim was to investigate covariates influencing the association between 5-year GH treatment and body composition in infants with FTT at the start of GH treatment, as well as the effect of age at treatment initiation. We hypothesized that infants with PWS and FTT_GH start_ who start GH treatment in the first year of life have more prominent effects on body composition during the first year of GH treatment compared to those starting later, between age 1 and 3 years.

## Methods

### Patients

All participants were diagnosed with PWS, confirmed by methylation analysis of the PWS region, and participated in the Dutch PWS Cohort study [11, 15, 16]. To investigate the effects of GH treatment, we included those with an age below 3.0 years at start of GH treatment who had at least 1 year of GH treatment at time of inclusion.

### Design

All participants were followed at the Dutch PWS Reference Centre in Rotterdam and received multidisciplinary care by the PWS team in collaboration with paediatric endocrinologists and paediatricians in other Dutch hospitals. Growth data preceding GH treatment were collected via questionnaires completed by the parents and via medical records. The Dutch PWS Cohort study was approved by the Medical Ethics Committee of the Erasmus University Medical Centre (MEC-2001-230, 19 September 2001). Written informed consent was obtained from parents. The study was conducted according to the guidelines of the Declaration of Helsinki II [17].

In the prospective GH study, all children were treated with 1.0 mg GH/m^2^/day (∼0.035 mg/kg/day). At each visit, GH dose was adjusted to their body surface area and serum IGF-1 levels were kept below +3 SDS. The mean GH dose was calculated for each patient as the sum of all annual dosages divided by the number of treatment years.

### Anthropometrics

Supine length was measured in centimetres with Harpenden Infantometer (Holtain Ltd., Crosswell, UK) and standing height using a Harpenden stadiometer. Measurements were performed by the PWS team. Body weight was measured in kilograms on a calibrated electronic digital scale (Servo Balance KA-20-150S; Servo Berkel Prior, Katwijk, The Netherlands). Birthweight, length, height, weight, and body mass index (BMI) standard deviation scores were calculated with Growth Analyser RCT 4.1 (available at www.growthanalyser.org), based on Dutch reference values [18, 19]. Small for gestational age was defined as birthweight and/or length below −2 standard deviation scores (SDS) [20]. We defined FTT as weight for length <−2 SDS [6]. Based on the growth data from birth to age 6 months, we determined the weight-for-length SDS at 1-, 3-, and 6-month time points, using linear interpolation. If GH treatment was initiated before the age of 6 months, these data were not included in the linear interpolation.

### Dual-Energy X-Ray Absorptiometry

DXA scans (Lunar Prodigy; GE Healthcare) were annually performed to measure LBM and fat mass percentage (FM%). The DXA machine was calibrated daily and all scans were made with the same machine. The intra-assay coefficients of variation were 0.41–0.88% for fat tissue and 1.57–4.49% for LBM [19]. LBM was calculated as fat-free mass minus bone mineral content. Fat percentage (FM%) was expressed as percentage of total body mass. LBMI was calculated as LBM (kg)/squared height (metres).

### Statistical Analysis

Statistical analyses were performed with SPSS version 27.0 (SPSS Inc. Chicago, IL). Clinical characteristics are presented as median with interquartile range (IQR) [6]. Mann-Whitney U tests, chi-square tests, and independent *t* tests were used to compare the baseline characteristics between the groups. Differences within groups were calculated using Wilcoxon signed-rank test and dependent *t* test. We used linear mixed-model analyses to assess changes over time, with LBMI, FM%, and weight-for-length SDS at each time point as dependent variables with an unstructured covariance matrix. Effects are presented as estimated marginal means (95% CI). The effects of FTT_GH start_ and age at start of GH treatment over time were investigated using these variables as factors (in case of nominal or ordinal variables) or covariates (in case of continuous variables) in the model. All linear mixed-model analyses included sex and age as covariates. Other potential covariates that were analysed were genotype, birthweight SDS, maternal BMI >25, and mean GH dose. Multiple linear regression analysis was used to determine the effect of FTT_GH start_ on LBMI and FM% at start and after 5 years of GH treatment. All multiple linear regression analyses included sex, age, and genotype as covariates. Other covariates that were analysed were birthweight SDS, maternal BMI >25, and mean GH dose.

A sub-analysis was performed in children with FTT_GH start_, comparing 2 subgroups based on the age at start of GH treatment: age at GH start between 0 and 0.99 years and age at GH start between 1 and 3 years. Differences were considered significant if the *p* value was <0.05.

## Results

### Percentage of Infants with FTT in the Period from Birth to Age 6 Months

Growth data from birth to age 6 months were obtained for 54 patients. Birthweight was available for 52 patients. Of these 52 patients, 10 (19.2%) were born small for gestational age. Median duration of tube feeding after birth was 23 (IQR 12, 32) weeks. In the period from birth to age 6 months, 29% had FTT at 1 month, 20% at 3 months, and 16% at 6 months (Fig. 1).

**Fig. 1.**
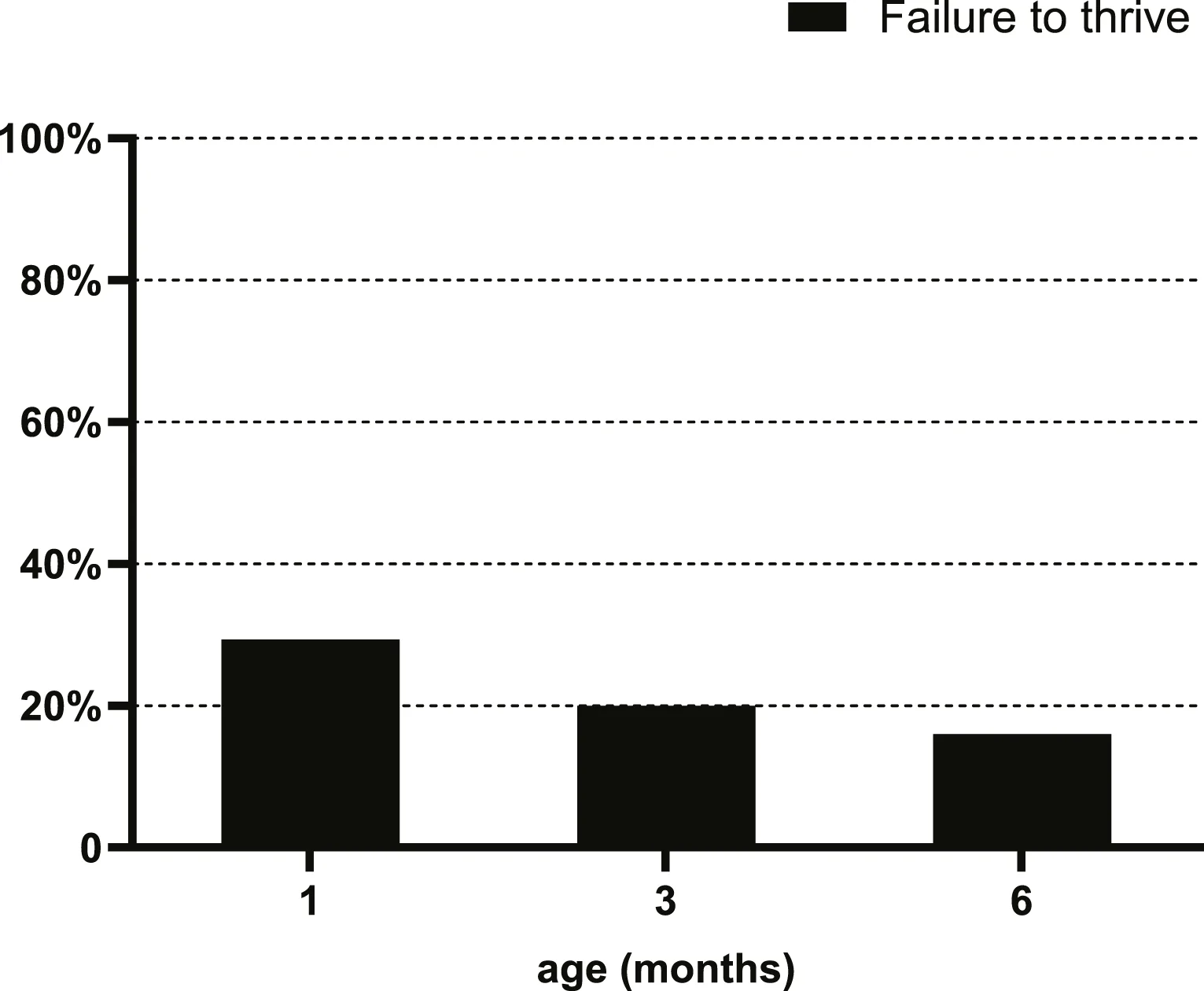
Percentage of infants with FTT among 54 infants with PWS from birth to 6 months. Percentage of infants with FTT among 54 infants with PWS from birth to 6 months of age, based on interpolated estimates. Sample sizes at each time point: 1 month (*n* = 41), 3 months (*n* = 50), 6 months (*n* = 38).

### Percentage of Infants with FTT at GH Start and Clinical Characteristics of Infants with and without FTT at GH Start

Table 1 shows the baseline characteristics at start of GH treatment. Twenty of the 166 (12%) infants had FTT at GH start (FTT_GH start_). Infants with FTT_GH start_ had a median age of 1.03 (IQR 0.45, 1.89) years at start of GH treatment and infants without FTT at GH start (non-FTT_GH start_) had a median (IQR) age of 0.69 (0.47–1.34) years (*p* = 0.22). No difference in genetic subtypes was found between the groups (*p* = 0.84). Infants with FTT_GH start_ had a median birthweight SDS of −1.03 (IQR −1.75, −0.28), compared to −0.95 (−1.60 to −0.34) in the non-FTT_GH-start_ group (*p* = 0.75). LBMI and FM% were lower in the FTT_GH-start_ compared to the non-FTT_GH-start_ group (*p* = 0.03 and *p* < 0.001, resp.). Length SDS and target height SDS were not different between the groups (0.34 and 0.29, resp.).

**Table 1.** Baseline characteristics at start of GH treatment

	FTT_GH start_	Non-FTT_GH start_	*p* value*
Number	20	146	
Sex (m/f)	14/6	79/67	0.18
Genetic subtype			0.84^a^
Deletion	12	85	
mUPD	7	57	
ICD	1	3	
Subtype unknown		1	
Age at start of GH treatment, years	1.03 (0.45–1.89)	0.69 (0.47–1.34)	0.22
Birthweight SDS	−1.03 (−1.74 to −0.28)	−0.95 (−1.59 to −0.34)	0.75
Weight-for-length SDS	**−2.34 (−2.94 to −2.15)**	**−0.25 (−0.97 to 0.50)**	**<0.001**
Weight SDS	**−3.14 (−4.02 to −2.60)**	**−1.39 (−2.45 to −0.49)**	**<0.001**
BMI SDS	**−2.70 (−3.20 to −2.22)**	**−0.64 (−1.28 to 0.39)**	**<0.001**
LBMI^b^	**10.05 (9.02–10.98)**	**10.79 (9.53–11.98)**	**0.03**
FM%^b^	**23.8 (21.4–31.7)**	**34.7 (28.6–38.9)**	**<0.001**
Length SDS	−1.73 (−2.71 to −0.69)	−1.43 (−2.25 to −0.59)	0.34

Data expressed as median (IQR). FTT, failure to thrive; FTT_GH start:_, infants who had FTT at GH start; mUPD, maternal uniparental disomy; ICD, imprinting centre defect; GH, growth hormone; BMI, body mass index, LBMI; lean body mass index.

*p value between group with FTT compared to group without.

^a^ Chi-square test was used to compare differences between groups.

^b^ *N* = 157.

In the FTT_GH-start_ group, 9 infants started GH treatment in the first year of life at a median age of 0.43 (IQR 0.34, 0.74) years and 11 infants between 1 and 3 years of age at a median age of 1.77 (IQR 1.11, 2.28) years. There was no difference in genotypes between subgroups. At start of GH treatment, infants who started GH treatment in the first year of life were taller (*p* = 0.02) and had a lower LBMI (*p* = 0.02) than infants who started GH treatment between 1 and 3 years of age. Target height SDS was not different between subgroups.

### Body Composition in the FTT_GH-Start_ versus Non-FTT_GH-Start_ Group during 5 Years of GH Treatment (*n* = 166)

Figure 2 shows the change over time (trajectories) of LBMI and FM% during 5 years of GH treatment in the FTT_GH-start_ group compared to the non-FTT_GH-start_ group, corrected for sex and age. At start of GH treatment, infants with FTT_GH start_ showed a lower LBMI compared to the non-FTT_GH-start_ group (*p* = 0.03). The trajectory of LBMI was also significantly different between the groups (*p* = 0.04). In both groups, an increase in LBMI was found after start of GH, but the increase in the FTT_GH-start_ group was greater in the first year, resulting in a similar LBMI after 5 years in both groups. Children with FTT_GH start_ still had a lower FM% compared to non-FTT_GH start_ after 5 years, while they had the same age. Genotype, birthweight SDS, maternal BMI >25, and mean GH dose were insignificant covariates.

**Fig. 2.**
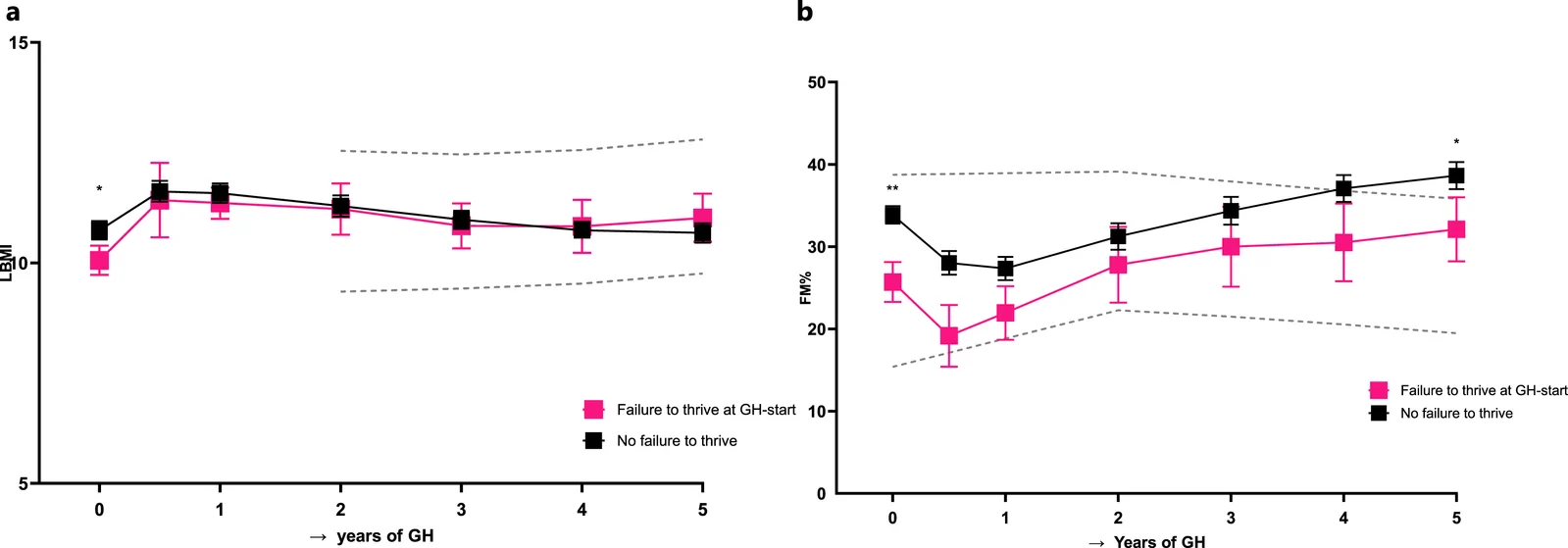
Trajectory of (**a**) LBMI and (**b**) FM% in infants with PWS with FTT_GH start_ and non-FTT_GH start_. Changes in body composition parameters in infants with PWS with or without FTT at GH start, corrected for sex and age, presented as estimated marginal means with 95% CI. Sample sizes at each year: 0 (*n* = 157), 1 (*n* = 142), 2 (*n* = 114), 3 (*n* = 114), 4 (*n* = 111), 5 (*n* = 97) years. *p value <0.05; **p value <0.001. Reference values of body composition data from healthy peers are presented as dotted lines (−2 and +2 SDS). Reference values for LBMI in children under 2 years of age are not available.

At start of GH treatment, infants who started GH treatment in the first year of life had a lower LBMI compared to infants who started GH treatment between 1 and 3 years of age (median LBMI 9.02 vs. 10.61, *p* = 0.02). There was no difference in FM% between these subgroups at GH start (0.20). The increase in LBMI in the first year of GH treatment was greater in the infants who started GH treatment in the first year of life compared to infants who started GH treatment between 1 and 3 years of age (+2.12 vs. +1.50, *p* = 0.02). After 5 years, there was no difference in LBMI and FM% between the subgroups (*p* = 0.55 and *p* = 0.55, resp.)

#### Multiple Regression Analyses

The multiple regression analyses results are shown in Table 2. At start of GH treatment, the presence of FTT_GH start_ was associated with a lower LBMI in multiple linear regression analysis, with sex, age, genotype, birthweight SDS, and maternal BMI >25 kg/m^2^ included in the model as covariates. After 5 years of GH treatment, FTT_GH-start_ was no longer associated with LBMI in the model including the same covariates. However, a higher IGF-1 SDS was associated with a higher LBMI after 5 years (B ± SE: 0.48 ± 0.20, *p* = 0.02). The mean GH dose tended to be associated with a higher LBMI after 5 years (B ± SE: 1.65 ± 0.82, *p* = 0.05).

**Table 2.** Multiple linear regression analysis at start of GH treatment and after 5 years

​	LBMI			FM%		
	B±SE	β	*p* value	B±SE	β	*p* value
GH start						
Sex	0.14±0.27	0.05	0.60	−1.72±1.45	−0.12	0.24
Age, years	1.06±0.21	0.49	<0.001	−4.45±1.16	−0.37	<0.001
Genotype	−0.35±0.26	−0.13	0.18	−3.13±1.41	−0.21	0.03
Birthweight SDS	−0.06±0.15	−0.04	0.70	2.91±0.79	0.35	<0.001
Maternal BMI >25	−0.20±0.30	−0.07	0.51	3.12±1.60	0.19	0.06
FTT_GH start_	−**1.22±0.39**	**−0.31**	**0.003**	**−4.45±2.14**	**−0.21**	**0.04**
Adjusted *R*^2^	0.323	​	<0.001	0.350	​	<0.001
After 5 years						
Sex	0.73±0.27	0.35	0.01	−2.66±2.11	−0.15	0.21
Age, years	0.44±0.23	0.25	0.06	−0.38±0.79	−0.03	0.83
Genotype	−0.19±0.26	−0.09	0.47	1.44±2.00	0.08	0.48
Birthweight SDS	0.19±0.15	0.16	0.20	1.79±1.16	0.18	0.13
Maternal BMI >25	0.85±0.34	0.33	0.02	7.63±2.66	0.35	0.006
Mean GH dose	1.65±0.82	0.82	0.05	−19.43±6.44	−0.38	0.004
FTT_GH start_	−**0.31±0.42**	**−0.10**	**0.47**	**−0.51±3.28**	**−0.02**	**0.88**
Adjusted *R*^2^	0.270	​	<0.001	0.370	​	<0.001

Results of multiple linear regression analysis. B ± SE, unstandardized coefficient and standard error; Β, standardized coefficient beta; FTT, failure to thrive; LBMI, lean body mass index; FM%, fat mass percentage; FTT_GH start_, infants who had FTT at GH start.

At start of GH treatment, the presence of FTT_GH start_ was associated with a lower FM%, with sex, age, genotype, birthweight SDS, and maternal BMI >25 kg/m^2^ included in the model as covariates. A higher birthweight SDS was associated with a higher FM%, and a maternal BMI >25 kg/m^2^ tended to be associated with a higher FM% (*p* < 0.001 and *p* = 0.06, resp.). After 5 years of GH treatment, FTT_GH start_ was no longer associated with FM% in the model including the same covariates. However, a higher mean GH dose was associated with a lower FM% (B ± SE: −19.43 ± 6.44, *p* = 0.004). IGF-1 SDS was not associated.

### Weight-for-Length Trajectory during 5 Years of GH Treatment

Since we defined FTT as weight for length < −2 SDS, we also investigated the changes in weight-for-length SDS over time. Estimated mean weight-for-length SDS decreased in the first 6 months of GH treatment in the total group, after which it significantly increased over the following 5 years (*p* < 0.001). The increase in weight-for-length SDS during the first 2 years of GH treatment was greater in the FTT_GH start_ compared to non-FTT group (+2.83 SDS vs. +0.81 SDS, *p* < 0.001). After 5 years of GH, weight-for-length SDS was 0.73 (−0.25–2.05) in the FTT_GH-start_ compared to 1.49 (0.70–2.53) in the non-FTT group (*p* = 0.06).

## Discussion

This study presents the percentage of infants with FTT in a group of 54 infants with PWS. It is the first study investigating the effect of GH treatment on body composition in 166 infants with PWS with FTT at GH start compared to those without FTT. From birth to age 6 months, 29% of infants had FTT at 1 month, 20% at 3 months, and 16% at 6 months. Twenty of the 166 (12%) infants had FTT at the start of GH treatment (FTT_GH start_). At GH start, infants with FTT_GH start_ had a lower LBMI compared to those without FTT_GH start_, followed by a greater catch-up in LBMI in the first year of GH treatment, resulting in a similar LBMI between the groups after 5 years of GH treatment. Children with FTT_GH start_ still had a lower FM% after 5 years of GH treatment compared to the non-FTT_GH-start_ group. However, after including covariates, FTT_GH start_ was no longer associated with FM% after 5 years.

Twenty-nine of the infants at 1 month, 20% at 3 months, and 16% at 6 months had FTT in the period from birth to age 6 months. The percentage of infants with FTT decreased with age, with most children having FTT at the age of 0–3 months. Twenty of the 166 (12%) infants had FTT at the start of GH treatment at a median age of 1.03 years. Few studies investigated the percentage of infants with FTT in infants with PWS. Miller et al. [3] described severe, prolonged FTT in 16% of the infants with PWS aged 0–9 months. In another study, 6 of the 9 (67%) infants, aged 10 days to 11 months, had a body weight below the 3rd percentile [9]. Singh et al. [21] described FTT in 77% of the babies with PWS, while another study described FTT in infants with PWS in a much higher prevalence of 94.8%, possibly due to parents’ refusal of tube feeding [22]. In these studies, the ages of the children varied and different definitions were used to diagnose FTT, or the definition was not specified, which may explain the differences in the reported percentages. Nowadays, Dutch children with PWS receive multidisciplinary care from early age and the patients in our study received tube feeding for a median duration of 23 weeks after birth, which may resolve FTT or prevents developing FTT.

Our data show that at GH start, infants with FTT_GH start_ had a lower LBMI compared with non-FTT_GH start_, but after 5 years of GH, LBMI was not different anymore between the groups due to a greater catch-up in LBMI in the FTT_GH-start_ group. This is in line with our previous finding that infants with a lower motor development had a greater improvement in motor development during GH treatment [12]. Our findings suggest that GH treatment has a greater impact on LBM development in infants with FTT than in those without FTT, indicating that the start of GH treatment should not be delayed until FTT is resolved.

The trajectory of LBMI showed a greater increase in infants with FTT_GH start_ who started GH in the first year of life compared to those with FTT_GH start_ who started GH between age 1 and 3 years. This corresponds to what we previously described in all infants with PWS, showing a greater and longer-term increase in LBMI in children who started GH in the first year of life compared to children who started between age 2 and 5 years [16]. This is particularly beneficial for young infants with PWS, given their hypotonia and motor developmental delay. A greater increase in LBMI will better support the motor development during early childhood.

Despite weight-for-length SDS remaining lower during 5 years in infants with FTT_GH start_ compared to non-FTT_GH start_, LBMI was similar between the groups after 5 years of GH treatment. This suggests that weight for length is not the best way to monitor FTT appropriately in infants with PWS. It is important to take this into account and to measure body composition when available.

This study adds to the limited literature by describing the percentage of FTT in 54 infants with PWS. In addition, we described the effects of GH treatment on body composition in 166 infants with PWS comparing infants with FTT at GH start to those without FTT. There were, however, some limitations. The subgroup of 54 infants represents only a limited sample, albeit considerable for a rare disorder like PWS. To study FTT in more detail, we need more data from birth to start of GH treatment. Due to the young age, LBMI and FM% SDS could not be calculated as reference values for body composition measurements by DXA scans are only available from 2 years of age.

In conclusion, our study shows that, 29% of infants had FTT at 1 month, 20% at 3 months, and 16% at 6 months. Twenty of the 166 (12%) infants still had FTT at the start of GH treatment. Children with FTT at GH start seem to benefit most from GH treatment in terms of LBM improvement, resulting in a similar LBM after 5 years of GH treatment as those without FTT at GH start.

## Acknowledgments

We express our gratitude to all children and parents for their enthusiastic participation in this study. We thank all collaborating paediatric-endocrinologists, paediatricians, and other health care providers.

## Statement of Ethics

The study was approved by the Medical Ethics Committee of the Erasmus Medical Centre/Sophia Children’s Hospital, Rotterdam, The Netherlands (PWS cohort study: protocol code MEC-2001-230, approved on 18 September 2001). Written informed consent to participate in this study was provided by the participants’ legal guardian/next of kin.

## Conflict of Interest Statement

Prof. Dr. Anita C. Hokken-Koelega was a member of the journal’s Editorial Board at the time of submission.

## Funding Sources

The authors did not receive any specific grant or funding for the work described in this article.

## Author Contributions

Demi Trueba-Timmermans, Anita Hokken-Koelega, Gerthe Kerkhof, Lionne Grootjen, and Edmond Rings contributed to this study. Demi Trueba-Timmermans has conducted the statistical analyses and drafted the manuscript, and Anita Hokken-Koelega, Gerthe Kerkhof, Lionne Grootjen, and Edmond Rings critically reviewed the manuscript. Demi Trueba-Timmermans, Anita Hokken-Koelega, Gerthe Kerkhof, Lionne Grootjen, and Edmond Rings read and approved the final manuscript.

## Data Availability

Data are not publicly available due to ethical reasons. Further inquiries can be directed to the corresponding author.
